# Identification of a Novel *Haemophilus parasuis*-Specific B Cell Epitope Using Monoclonal Antibody against the OppA Protein

**DOI:** 10.1371/journal.pone.0084516

**Published:** 2014-01-09

**Authors:** Nan Zheng, Zheng Chai, Fang Fu, Fucheng Jiang, Xiangling Wang, Xueyun Zhang, Zhuo Wang, Xi Li

**Affiliations:** State Key Laboratory of Veterinary Biotechnology, Harbin Veterinary Research Institute, the Chinese Academy of Agricultural Sciences, Harbin, China; National Cancer Institute, NIH, United States of America

## Abstract

Monoclonal antibody (MAb) 1B3 against *Haemophilus parasuis* (*H. parasuis*) was generated by fusing SP2/0 murine myeloma cells and spleen cells from BALB/c mice immunized with the whole-bacterial-cell suspension of *H. parasuis* HS80 (serotype 5). The MAb 1B3 showed strong reactivity with 15 serotype reference strains of *H. parasuis* using Dot blot and Western blot analysis. Immunoprecipitation and protein spectral analysis indicated that MAb 1B3 recognized by Oligopeptide permease A (OppA) belongs to the ATP binding cassette transporter family. In addition, a linear B-cell epitope recognized by MAb 1B3 was identified by the screening of a phage-displayed 12-mer random peptide library. Sequence analysis showed that MAb 1B3 was recognized by phages-displaying peptides with the consensus motif KTPSEXR (X means variable amino acids). Its amino acid sequence matched ^469^KTPAEAR^475^ of *H. parasuis* OppA protein. A series of progressively truncated peptides were synthesized to define the minimal region that was required for MAb 1B3 binding. The epitope was highly conserved in OppA protein sequences from the isolated *H. parasuis* strains, which was confirmed by alignment analysis. Furthermore, the minimal linear epitope was highly specific among 75 different bacterial strains as shown in sequence alignments. These results indicated MAb 1B3 might be potentially used to develop serological diagnostic tools for *H. parasuis*.

## Introduction

*Haemophilus parasuis* (*H. parasuis*) is a Gram-negative, nonhemolytic, NAD-dependent bacterium belonging to the *Pasteurellaceae* family. This bacterium is the causative agent of Glässer's disease. Its main symptoms include pericarditis, polyarthritis, multiple fibrinous serositis and meningitis [1]. Glässer's disease leads to high morbidity and mortality in non-immune pigs and inflicts severe economic loss in the pig industry. In recent years, *H. parasuis* has become an important pathogen in the pig industry all over the world [1], [2].

The identification of *H. parasuis* has traditionally been accomplished by culture isolation and biochemical analysis [3]. To date, 15 serotypes of *H. parasuis* have been described, but up to 25% of the isolates in some countries cannot be typed [4]. The most popular serological technique is immunodiffusion [5], [6] or indirect hemagglutination [7]. Antibodies with a high affinity and specificity for bacterial protein could be used to detect the pathogens by immunological methods. With such high quality antibodies in conjunction with the advent of new technologies, cultural enrichment may be necessary for the detection.

Detailed analysis of the epitope plays an important role in the understanding of immunological events and the development of epitope-based diagnostic tools for various diseases [8]–[10]. In this study, we described the generation and characterization of a monoclonal antibody 1B3 that reacted with 15 serotypes of *H. parasuis*. Importantly, we used immunoprecipitation, MALDI-TOF-MS and phage-displayed random 12-mer peptide library to determine whether MAb 1B3 could recognize the linear B-cell epitope located on the OppA protein of *H. parasuis*. The provided information in this study will facilitate the development of specific serological diagnosis of *H. parasuis* infection.

## Materials and Methods

### Ethics statement

This study was carried out in strict accordance with animal ethics guidelines and approved protocols. All animal studies were approved by the Animal Ethics Committee of Harbin Veterinary Research Institute of the Chinese Academy of Agricultural Sciences (SYXK (H) 2006-032).

### Bacterial strains and culture media

The reference strains of *H. parasuis* (strains 1 to 15) were kindly supplied by Xiaoling Chen from Beijing Academy of Agriculture and Forestry Science, China. The reference strains of *Salmonella enterica* (*S. enterica*), *Staphylococcus* aureus (*S. aureus*), enterotoxigenic *Escherichia coli* (ETEC) and *Actinobacillus pleuropneumoniae* (*A. pleuropneumoniae*) were acquired from the State Key Laboratory of Veterinary Biotechnology, Harbin Veterinary Research Institute. The reference *H. parasuis* HS80 strain (serotype 5) was used for the production of monoclonal antibody. These bacteria were propagated by standard techniques. *H. parasuis* was maintained on tryptic soy agar (TSA, BD) containing 10% bovine serum and 0.01% NAD or cultured aerobically in tryptic soy broth (TSB, BD) plus 10% bovine serum and 0.01% NAD at 37°C.

### Production and characterization of *H. parasuis*-reactive monoclonal antibody

*H. parasuis*-specific MAbs were produced as follows. Briefly, 6-week-old female BALB/c mice were immunized with 100 µg *H. parasuis* mixed with complete Freund's adjuvant (Sigma) per mouse. Two booster injections containing *H. parasuis* with an equal volume of Freund's incomplete adjuvant were conducted in a two-week interval. Two weeks later after injection, the mice were intraperitoneally boosted with 100 µg *H. parasuis*. After three days, splenocytes were fused with SP2/0 myeloma cells using polyethylene glycol (PEG 4000; Sigma). The procedure was performed as previously described [11], [12]. Following fusion, the cells were diluted into 96-well plates and cultured in hypoxanthine-aminopterin-thymidine (HAT) selection medium. After 6 days, the medium was removed and replaced with hypoxanthine-thymidine (HT)-DMEM medium. After selection in HAT or HT media, hybridomas were screened for reactivity and specificity by indirect enzyme-linked immunosorbent assay (ELISA). This approach produced one *H. parasuis*-reactive MAb that was named as MAb 1B3. The isotype of MAb 1B3 was determined by SBA Clonotyping™ System (Southern Biotech, USA). Eight-week old BALB/c mice were injected intraperitoneally with hybridoma 1B3 and ascite fluid was collected and purified using HiTrap Protein G HP (Millipore, USA).

### Indirect ELISA

Plates were coated with 0.4 µg/well of *H. parasuis* lysates diluted in carbonate-bicarbonate buffer (pH 9.6) at 4°C overnight. The coated plates were blocked with 5% (w/v) skim milk in PBST (1× PBS with 0.05% Tween 20) and then incubated with the supernatant of hybridoma culture and HRP-conjugated goat anti-mouse IgG antibody (Sigma, USA) for 1 h at 37°C. TMB substrate (Tian Gen, China) was added for colorimetric detection. The results were analyzed using a spectrophotometer at an absorbance of 450 nm.

### Western blot and Dot blot

The lysates of all 15 serotype reference strains of *H. parasuis*, and other four species were subjected to the analysis of 12% sodium dodecyl sulfate polyacrylamide gel electrophoresis (SDS-PAGE). For Western blot, proteins in the gel were transferred to nitrocellulose membrane that was then blocked with 5% skimmed milk diluted in PBS. Membranes were incubated with the hybridoma 1B3 culture supernatants for 1 h. Subjected to 3 times washes, the membranes were incubated with horseradish peroxidase (HRP)-conjugated goat anti-mouse IgG for 1 h. Finally, the membrane was washed and incubated with 3, 3-Diaminobenzidine tetrahydrochloride as the substrate to visualize the reaction result. The Dot blot procedure was performed using 10 µL of each sample. Protein denaturation reagents were omitted during sample preparation to preserve the native conformation in the Dot blot. The further procedures were as Western blot described.

### Immunoprecipitation and MALDI-TOF-MS

Bacterial cells were re-suspended in PBS-1% Triton X-100 for 1 h at 4°C. Samples were pre-cleared with protein A-coated agarose beads for 30 min, followed by incubation with MAb for 1 h at 4°C. Totally 20 µL of immunoprecipitate was re-suspended with electrophoresis sample buffer. Samples were boiled, and then analyzed by SDS-PAGE. MAb 1G7, specifically bound to the N protein of porcine reproductive and respiratory syndrome virus, was used as a negative control for monoclonal antibody [13]. ETEC was used as a negative control antigen.

The immunoprecipitates were washed three times for 10 min at 4°C with washing buffer 1 (0.1% N-octylglucoside, 140 mM NaCl, 10 mM Tris pH 8.0, 0.025% sodium azide) and once with washing buffer 2 (10 mM Tris pH 8.0, 0.025% sodium azide). Then, the immunoprecipitates were eluted with trifluoroacetic acid/acetonitrile/water (1∶20∶20) saturated with alpha-cyano-4-hydroxy cinnamic acid. The dissolved samples were dried on a stainless plate and subjected to MALDI-TOF MS analysis. Mass spectra were acquired by using an Ettan MALDI-TOF Pro mass spectrometer (GE Healthcare) by Sensichip Infortech Co. Ltd (China).

### Biopanning and phage ELISA

A commercially available Phage Display Peptide Library Kit was purchased from New England BioLabs Inc.. Three rounds of biopanning were carried out according to the manufacturer's instruction manual. Briefly, a 96-well plate was coated with 100 µg/mL purified MAb 1B3 in 0.1 M NaHCO_3_ buffer (pH 8.6) and incubated overnight at 4°C. The plate was washed with blocking buffer (0.1 M NaHCO_3_, 0.02% NaN_3_, 1% BSA and pH 8.6) for 2 h at 4°C. Approximately 2×10^11^ phages in the phage library (M13) were incubated for 1 h at room temperature. The plate was then washed ten times with TBS buffer (50 mM Tris-HCl, pH 7.5, 150 mM NaCl) containing gradually increased concentrations (0.1%, 0.3% and 0.5%) of Tween-20 (TBST). Bound phage was eluted with 100 µL of 0.2 M glycine/HCl (pH 2.2) containing 1 mg/mL BSA and the eluate neutralized with 15 µL of 1 M Tris-HCl (pH 9.1). The eluted phages were amplified in *E. coli* (ER2738), and titrated on Luria-Bertani (LB) medium plates containing isopropy-β-D-thiogalactoside (IPTG) and X-Gal plates for the subsequent rounds of selection. Fifteen individual phage clones derived from the third round of biopanning were selected for target binding in ELISA as described [10], [14]. Eight single-stranded DNA was prepared and sequenced by using the −96 sequencing primer (5′-CCCTCATAGTTAGCGTAA-3′). Inserted oligonucleotide sequences of phage DNA were selected and translated to peptide sequences.

### Expression of truncated OppA protein in *E. coli*

According to the encoding sequence of *H. parasuis* OppA protein (GenBank accession No. ACL32731.1), a pair of primers was designed to amplify a 288 bp fragment (Forward: 5′-AAAGGATCCTCAGTATTCGGTAACGACTTAGA-3′, Reversed: 5′-AAACTCGAGACCTGTGGTTGATAATGGTT-3′). The fragment was cloned into pET32a (+) vector (Novagen, USA) to construct the recombinant plasmids. The expression plasmid was transformed into BL21 (DE3) competent cells (Takara, China), followed by the addition of 1 mM IPTG (GE Healthcare, USA) for induction.

### Peptide design and epitope identification

In order to verify whether MAb 1B3 can recognize the corresponding epitope in *H. parasuis*, eight peptides were synthesized with the linkage of the irrelevant peptide “HHHHHHHHHHHHHHH” as the peptide control (Table 1). Then, the reactivity of the polypeptide with MAb 1B3 or the murine antisera was performed by ELISA.

**Table 1:** Progressively truncated peptide sequences for defining the minimal linear epitope.

Peptide Designation	Peptide Sequence^a^
1B3-1	*HHHHHHHH*KTPSEAR
1B3-2	*HHHHHHHHH*TPSEAR
1B3-3	*HHHHHHHHHH*PSEAR
1B3-4	*HHHHHHHHH*KTPSEA
1B3-5	*HHHHHHHHHH*KTPSE
1B3-6	*HHHHHHHH*HHHHHHH
1B3-7	*HHHHHHHH*KTPSEHR
1B3-8	*HHHHHHHH*KTPAEAR

^a^ Seven polypeptides were synthesized and the irrelevant peptide “HHHHHHHHHHHHHHH” was a peptide control.

In order to confirm the mapped epitope recognized by MAb 1B3 in *H. parasuis*, a competitive inhibition ELISA was performed. The 96-well plates were coated with 10 µg/well whole-cell lysates of *H. parasuis* HS80 strain in 0.1 M NaHCO_3_ (pH 8.6) at 4°C overnight, and then blocked with 5% skimmed milk diluted in PBS for 1 h at 37°C. Serial dilutions of synthetic peptides were pre-incubated separately with MAb 1B3 (0.5 µg/mL) for 1 h at 37°C. The antibody-peptide mixture was incubated for 20 min at 37°C. The HRP-conjugated goat anti-mouse antibody (1∶5000) was added for 1 h after the plates were washed five times. TMB substrate (Tian Gen, China) was added for colorimetric detection. The results were analyzed using a spectrophotometer at an absorbance of 450 nm.

### Homology analysis

In order to evaluate the specificity of the identified linear OppA epitope among *H. parasuis* strains, we aligned the sequences corresponding to the region encompassing the 1B3 peptide epitope from *H. parasuis* OppA protein sequences from GenBank using MEGA4.0. In addition, 75 sequences of bacterial OppA proteins from UNIPROT database were aligned by ClustalW method using the Genious 5.6.5 software to evaluate the conservation of mapped epitopes.

### Statistical analysis

The experimental data were analyzed by GraphPad Prism Version 5.01 for Windows (GraphPad Software, USA), and expressed as Mean ± SD.

## Results

### Generation and characterization of monoclonal antibody against *H. parasuis*

**Figure 1:**
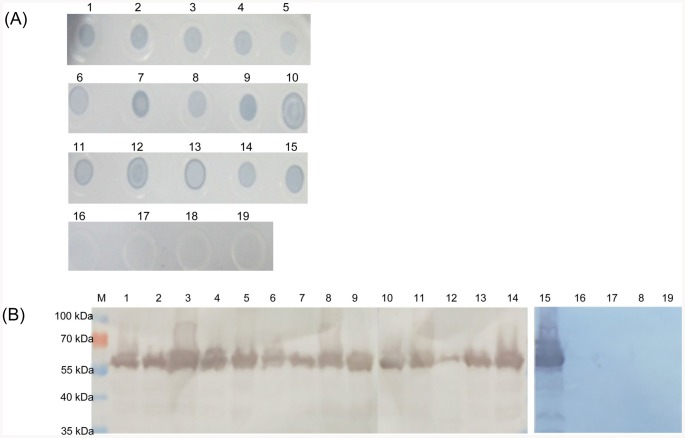
The binding of MAb 1B3 with all 15 reference strains of *H. parasuis*. (A) Reactivity of MAb 1B3 with the lysates of all 15 serotype strains of *H. parasuis* by Dot blot analysis. 1–15: 15 serotype reference strains of *H. parasuis*, respectively; 16: *S. enterica*; 17: *S.* aureus; 18: ETEC; 19: *A. pleuropneumoniae*. (B) Reactivity of MAb 1B3 with the lysates of all 15 serotype strains of *H. parasuis* by Western blot analysis. M: PageRuler™ Prestained Protein Ladder; Lane 1–15: 15 serotype reference strains of *H. parasuis*, respectively; Lane 16: *S. enterica*; Lane 17: *S.* aureus; Lane 18: ETEC; Lane 19: *A. pleuropneumoniae*.

The hybridoma 1B3 was generated by the fusion of murine myeloma SP2/0 cells with splenocytes from mice immunized with *H. parasuis*. The MAb 1B3 was composed of an IgG1 heavy chain paired with a κ light chain. The titers of antibody in hybridoma cell culture supernatants and in ascites were measured by indirect ELISA and determined to be 1∶800 and 1∶40,000, respectively. Dot blot was used to evaluate the specificity of MAb 1B3. The other bacteria such as *S. enterica*, *S.* aureus, ETEC and *A. pleuropneumoniae* were used as the negative controls. As shown in Figure 1A, MAb 1B3 reacted with all 15 serotype reference strains of *H. parasuis*, but failed to react with *S. enterica*, *S. aureus*, ETEC and *A. pleuropneumoniae*. We further characterized the physical properties of the recognized antigen by MAb 1B3 using Western blot analysis. A broad band with an approximate molecular mass of 58 kDa was observed (Figure 1B).

### Identification of bacterial protein reacting with monoclonal antibody

**Figure 2:**
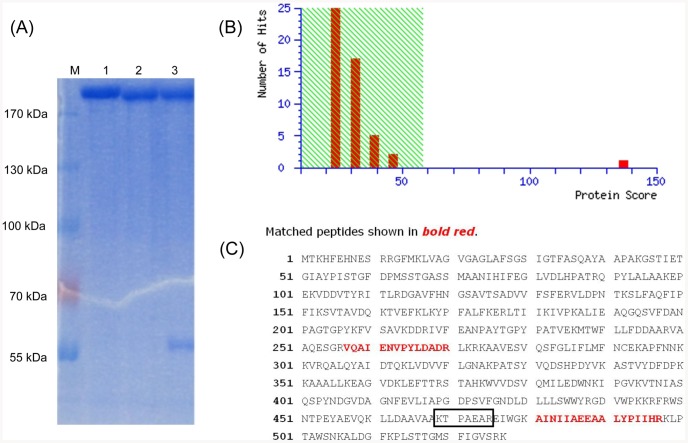
Identification of bacterial protein reacting with MAb 1B3. (A) SDS-PAGE of bacterial proteins after IP. The MAb 1G7-HPS HS80 mixture and the MAb 1B3-ETEC mixture were used as the negative controls. Lane 1: MAb 1G7-HPS HS80; lane 2: MAb 1B3-ETEC; lane 3: MAb 1B3-HPS HS80. (B) Identified results of protein spots through MALDI-TOF-MS. Protein scores (n = 152 for MAb 1B3) that were greater than 58 were significant (*P*<0.05). Protein scores were derived from ion scores as a non-probabilistic basis for ranking protein hits. (C) The amino acid sequence of OppA. The matched peptides with the OppA protein sequence were shown in bold red.

In order to determine bacterial protein that can bind to MAb 1B3, *H. parasuis* lysates were subjected to immunoprecipitation (IP) with MAb 1B3. Then, the immunoreactive protein band was shown in the Coomassie-stained gel (Figure 2A). The immunoprecipitates were analyzed by MALDI-TOF-MS on the basis of peptide mass matching. By searching the NCBI database, the protein (protein scores 136 larger than 58 were significant, *P*<0.05) that reacted with MAb 1B3 was identified as OppA protein, which was a protein with a molecular mass of 58 kDa (Figure 2B). Figure 2C shows the identified sequence with the coverage of 5%.

### Phage-displayed mimic epitope biopanned by MAb 1B3

To determine the epitope recognized by MAb 1B3, biopanning of a phage displayed 12-mer random peptide library was performed using the affinity of purified MAb 1B3. After three rounds of biopanning, an enrichment of phages that bound to MAb 1B3 was achieved. The output to input ratio following each of three rounds of biopanning was 0.00018%, 0.024% and 0.89%, respectively.

**Figure 3:**
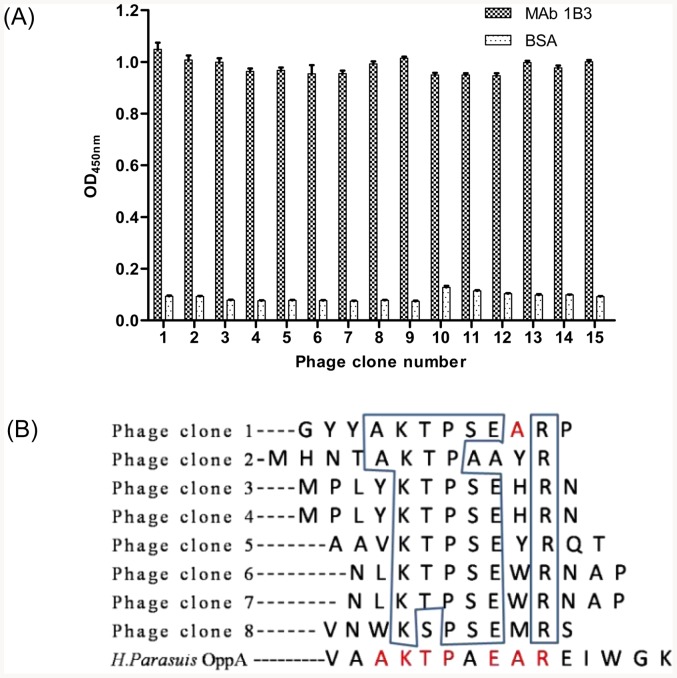
Identification of the epitope recognized by MAb 1B3. (A) Detection of the selected phages for antibody binding by Phage ELISA. Fifteen phage clones selected after three rounds of biopanning were analyzed the binding activity to MAb 1B3 by Phage ELISA. Three independent assays were carried out. (B) Sequence comparison of random peptide inserts displayed on the positive phages. The conservative amino acid motifs are boxed.

Fifteen phage clones were isolated. Their reactivity was evaluated through MAb 1B3 by phage ELISA. BSA was used as the negative control. As shown in Figure 3A, all selected fifteen phage clones reacted with MAb 1B3 (OD_450 nm_>0.8), but not with the negative control BSA (OD_450 nm_<0.2). The eight phage clones were sequenced, and revealed a consensus sequence of KTPSEXR (X means variable amino acids) (Figure 3B). The conserved sequence motif defined by the peptide library screen was highly similar to the sequence ^469^KTPAEAR^475^ observed in OppA protein of *H. parasuis* (Figure 3B).

### Localization and minimal reactivity unit of the epitope recognized by MAb 1B3

**Figure 4:**
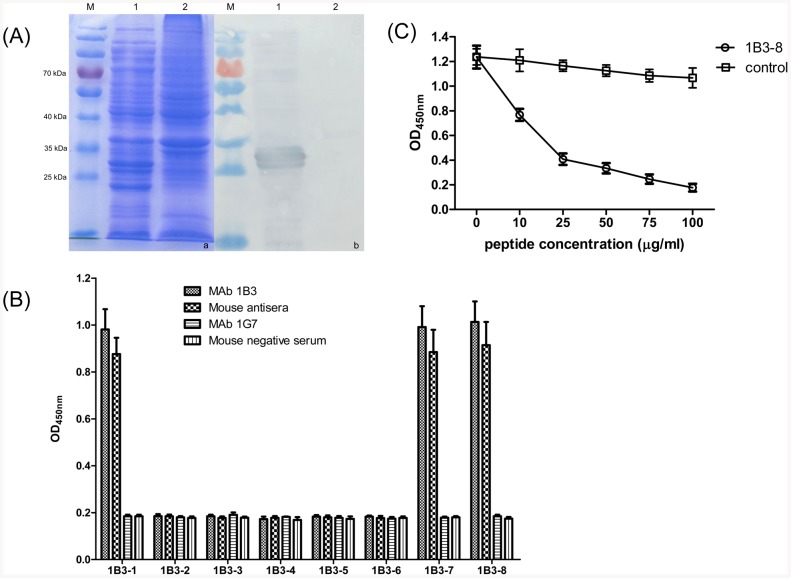
Confirmation of the epitope recognized by MAb 1B3. (A) Reactivity between MAb 1B3 and the truncated OppA protein of *H. parasuis*. (a) Coomassie brilliant blue stained SDS-PAGE of the truncated OppA protein expression. (b) Reactivity of the truncated OppA protein with MAb 1B3 by Western blot analysis. M: PageRuler™ Prestained Protein Ladder; Lane 1: pET-OppA; Lane 2: empty vector pET-32a. (B) Progressively truncated peptides defined the minimal linear epitope recognized by MAb 1B3 or murine antisera. MAb 1B3 was screened against a series of truncated peptides with progressively deleted amino acid residues from the amino and/or carboxyl termini to determine the minimal linear peptide sequence required for MAb 1B3 binding. (C) Competitive inhibition assay using increasing concentrations of the peptide 1B3-8 and control peptide 1B3-6.

To determine whether MAb 1B3 can react with OppA protein of *H. parasuis*, SDS-PAGE and Western blot showed that the truncated OppA protein with the molecular mass of approximately 28 kDa was expressed and reacted with MAb 1B3 (Figure 4A).

To precisely identify the epitope recognized by MAb 1B3, a panel of polypeptides was synthesized in which amino acids were progressively deleted from the amino and/or the carboxyl terminus of the ^469^KTPAEAR^475^ peptides screened by the phage display peptide library. The full-length of KTPAEAR peptide (1B3-8) was recognized by MAb 1B3, while the removal of one or more amino acids at either the carboxyl or amino terminus of the peptide resulted in the complete abrogation of antibody binding (Figure 4B). Furthermore, the polypeptide 1B3-7 with an A474H of the OppA protein still showed a good reactivity, indicating that the amino acid residues at the position of 474 was not important for the binding between the epitope and MAb 1B3 (Figure 4B). These results suggested that ^469^KTPAE-R^475^ is the minimal requirement for the reactivity of the epitope with MAb 1B3. In addition, as shown in Figure 4C, the competitive inhibition ELISA revealed a dose-dependent manner as the increase of peptide concentrations. When the concentration of MAb 1B3 was 0.5 µg/mL, the binding of MAb 1B3 to *H. parasuis* was significantly inhibited by the synthetic peptide 1B3-8 (Figure 4C). The inhibition was enhanced with the increasing concentration of the synthetic peptide. These results indicated that the peptide KTPAEAR is the epitope of *H. parasuis*, which is recognized by MAb 1B3.

### Homology analysis

**Figure 5:**
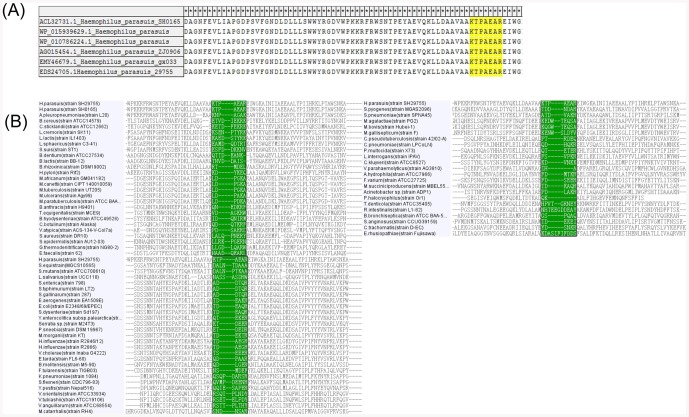
Amino acid sequence alignments of the epitope regions from the *H. parasuis* OppA protein. (A) Sequence alignments of 6 *H. parasuis* strains from GenBank. The GenBank accession numbers of *H. parasuis* strains are shown in front of the strains' names. The homologous sequences of different *H. parasuis* strains corresponding to the identified epitope are highlighted. (B) Sequence alignments of the epitope region from OppA proteins of *H. parasuis* and other 75 animal bacteria. The sequence corresponding to the region encompassing *H. parasuis* minimal linear epitope was identified and aligned for a panel of 75 bacterial strains from animals. The defined epitope and their corresponding regions in OppA proteins of bacteria are highlighted.

To investigate the conservation of the KTPSEAR epitope, we aligned the identified epitope with *H. parasuis* OppA protein encoding the regions available in GenBank. The alignment result showed that the epitope was identical among all OppA proteins of *H. parasuis* (Figure 5A), indicating that the motif was a conserved epitope in the OppA protein of *H. parasuis*.

To evaluate whether the conserved epitope of *H. parasuis* was specific among 75 animal bacteria, the amino acid sequences were aligned with corresponding region encompassing *H. parasuis* OppA minimal linear epitope from 75 animal bacteria. As shown in Figure 5B, the conserved epitope was highly specific among different bacterial strains.

## Discussion

*H. parasuis* is a widespread major pathogen that affects naive herds and causes a variety of production loss, including nursery mortality, decreased body weight gain, and lower meat value at slaughter [15]. Due to the limitation of knowledge regarding to the pathogenesis, virulence factors and immunogenicity of *H. parasuis*, it is difficult to control its systemic infection in pigs [16], [17]. Mapping epitopes with recognition function by monoclonal antibodies is a powerful tool to diagnose diseases [19]. The previous studies have reported the generation and characterization of several MAbs against outer member protein of *H. parasuis* [17], [18]. However, they only identified the relative regions recognized by MAbs, and did not map the precise locations. Based on our knowledge, no report on linear epitope mapping of *H. parasuis* is available. In the present study, we described the generation and epitope mapping of *H. parasuis* species-specific MAb 1B3, and identified the conserved epitopes among *H. parasuis* without reactivity with other bacteria. Precise analysis of the epitope will provide the fundamental information for the development of diagnostic tools for *H. parasuis* and other bacteria.

In this study, we also generated a *H. parasuis*-specific MAb 1B3 using the whole bacteria as an immunogen to ensure the integrity of the bacterial protein. Dot blot and Western blot analysis indicated that MAb 1B3 reacted with all 15 reference strains of *H. parasuis*, but not with other non-*H. parasuis* bacteria, such as *S. enterica*, *S.* aureus, ETEC and *A. pleuropneumoniae* (Figure 1). These results suggested that MAb 1B3 might be recognized a species-specific epitope, which could be used to distinguish *H. parasuis* from other bacteria.

Immunoprecipitation (IP) is a useful tool for the quick and simple purification of a target molecule from biological samples such as cell lysates, serum, plasma, and cerebrospinal fluid. Meanwhile, IP is an essential purification step prior to mass spectrometry (MS) analysis of low abundance peptides in biological fluids, because interference from non-target molecules should be avoided in MS analysis [20]. We used MAb 1B3 for immunoprecipitation to capture the specific bacterial protein (Figure 2A), which avoided the interference of non-target proteins and laid a good foundation for mass spectrometry analysis. IP-MS analysis indicated that the immunogenic target region of MAb 1B3 was located in OppA protein of *H. parasuis* (Figure 2). OppA belongs to the ATP-binding cassette transporter, which has been identified previously as the target for the development of vaccines against pathogenic bacteria [21]–[23]. However, there is no report for the *H. parasuis* OppA protein.

The linear epitope recognized by MAb 1B3 was defined as KTPSEXR (X means variable amino acids) using phage display technology to perform a screen of a peptide library. This peptide sequence directly corresponded to the sequence ^469^KTPSEAR^475^ of OppA protein (Figure 3B). Further precise mapping using synthesized peptides revealed that the core determinant of the MAb 1B3-binding site was ^469^KTPSE-R^475^ (Figure 4). The reactivity between the peptide and *H. parasuis*-positive mouse serum indicated that the epitope was immunogenic in mouse in the context of bacterial infection, suggesting that the OppA protein might be an immunogen to vaccine for *H. parasuis* infection. In addition, sequence alignments of OppA demonstrated that the motif was highly conserved among *H. parasuis* strains, suggesting that the epitope is a broad species-specific epitope. Furthermore, we found that the *H. parasuis* OppA minimal linear epitope was specific among 75 animal bacteria using sequence alignment analysis. The linear epitope was highly specific among different bacterial strains, suggesting that it could be developed a differential diagnostic method for *H. parasuis* and other animal bacteria.

In summary, we developed a *H. parasuis* specific-MAb 1B3 and defined the highly conserved linear B-cell epitope within OppA protein due to the recognition by MAb 1B3. The *H. parasuis* species-specific MAb and its defined linear epitope could be used for the development of a novel diagnostic method to distinguish *H. parasuis* from bacterial pathogens.
